# Supplementary material for: Analysis of Electrochemical Performance with Dispersion Degree of CNTs in Electrode According to Ultrasonication Process and Slurry Viscosity for Lithium-Ion Battery
Source: Nanomaterials (Basel). 2022 Dec 1;12(23):4271. doi: 10.3390/nano12234271 (PMC9735946; doi:10.3390/nano12234271)
Supplement: Supplementary file 1 [file nanomaterials-12-04271-s001.zip › nanomaterials-2052655-supplementary.pdf]

Supporting Information

Analysis of Electrochemical Performance with Dispersion Degree of CNTs in Electrode according to Ultrasonication Process and Slurry Viscosity for Lithium-Ion Battery

Jaehong Choi [1], Chaewon Lee [1], Sungwoo Park [2], Tom James Embleton [1], Kyungmok Ko [1], Mina Jo[1], Kashif Saleem Saqib [1], Jeongsik Yun [1], Minki Jo [3], Yoonkook Son [3] and Pilgun Oh [1,2,*]

[1] Department of Smart Green Technology Engineering, Pukyong National University, 45, Yongso-ro, Nam-gu, Busan 48547, Republic of Korea
[2] Department of Nanotechnology Engineering, Pukyong National University, 45, Yongso-ro, Nam-gu, Busan 48547, Republic of Korea
[3] Department of Electrical Engineering, Chosun University, 309, Pilmun-daero, Dong-gu, Gwangju 61452, Republic of Korea
* Correspondence: poh@pknu.ac.kr; Tel.: +82-51-629-6387; Fax: +82-51-629-6388

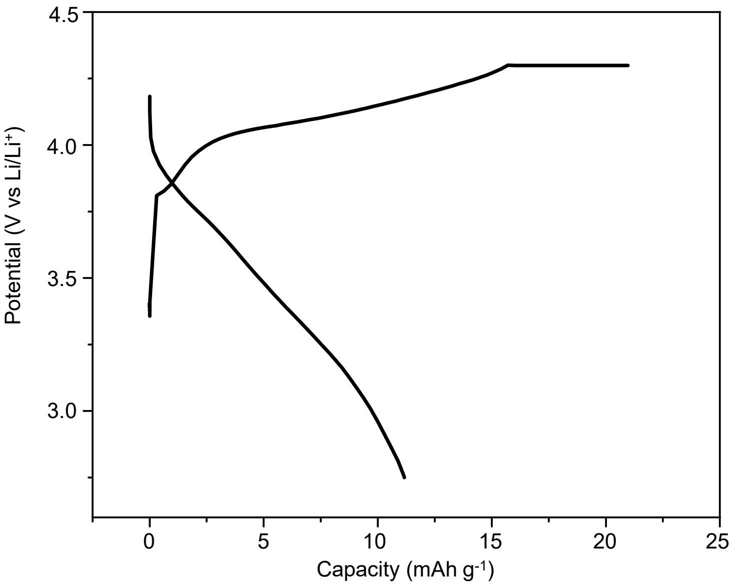

Figure S1. Voltage profile at 0.1C showing a cell without the application of a carbon additive. The lack of electrical contact heavily limits the cell to extremely low charge and discharge capacities. Cycle data is not available due to the poor performance.

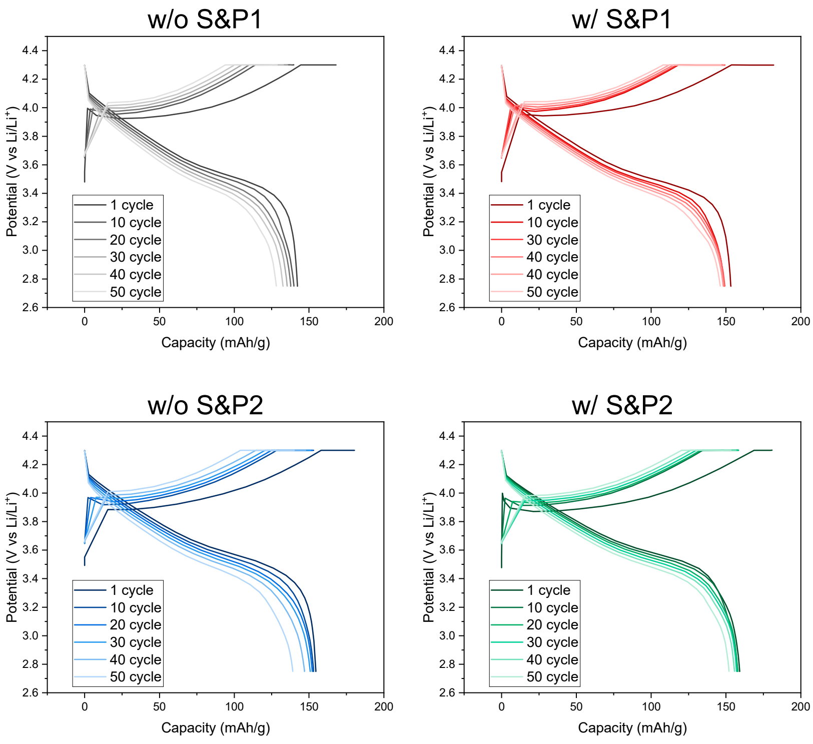

Figure S2. Comparison of voltage profiles of the 1,10,20,30,40 and 50 cycle data for w/o S&P1, w/ S&P1 , w/o S&P2, and w/ S&P2. The curves come from the cycle data in Figure 3.

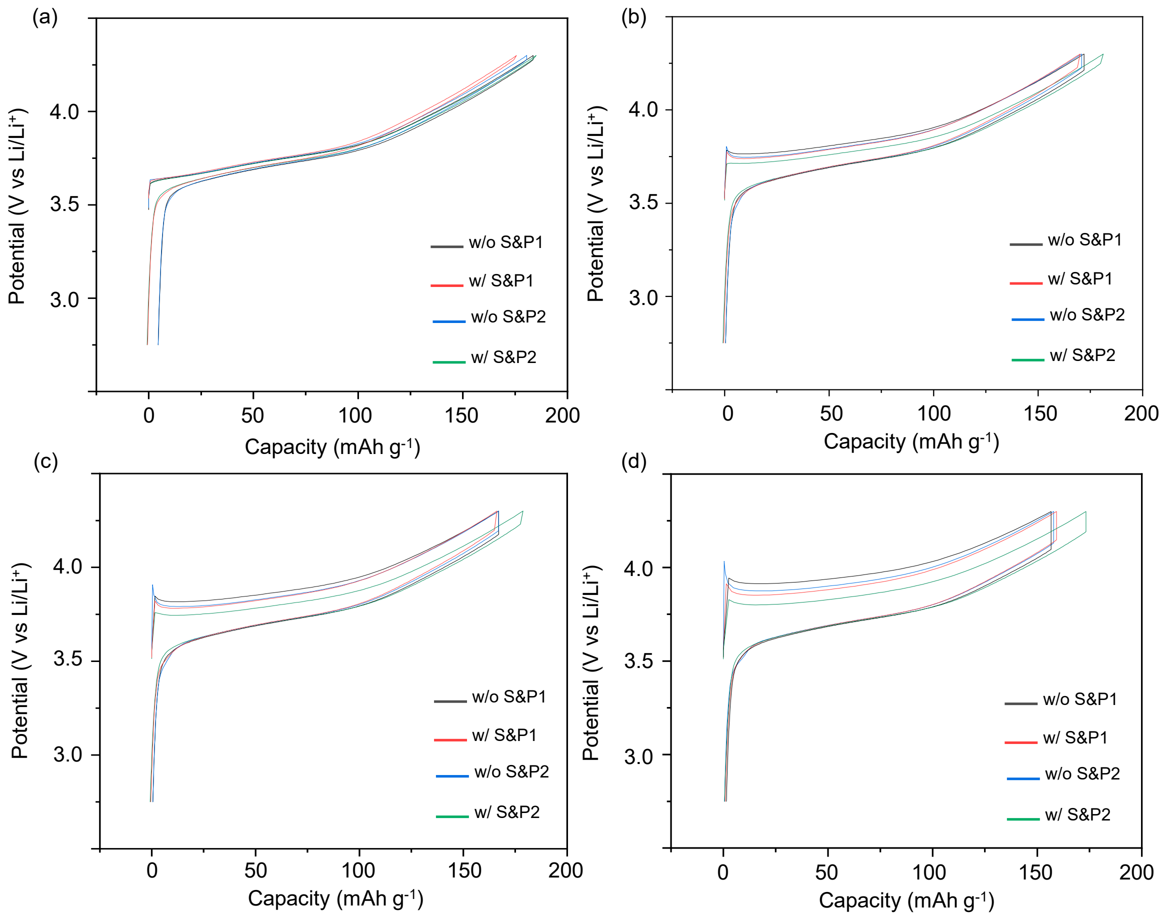

Figure S3. Comparison of voltage profiles of the first cycle data for (a) 0.2C, b) 2C, c) 3C, and d) 5C showing charge and discharge cycles. The curves come from the rate data in Fig. 4(a).

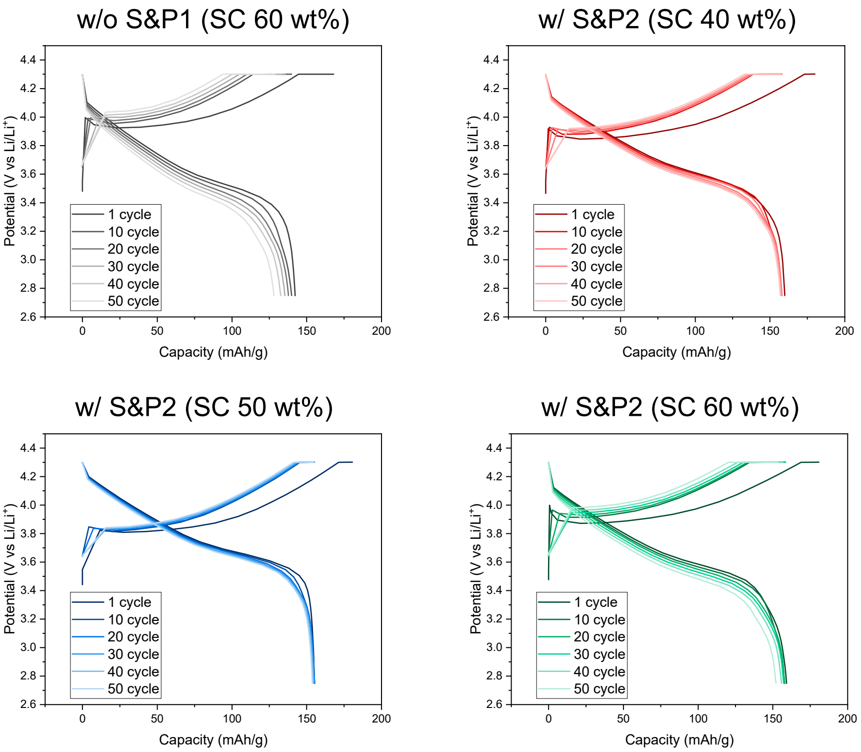

Figure S4. Comparison of voltage profiles of the 1,10,20,30,40 and 50 cycle data for w/o S&P1, w/ S&P2 (SC 40 wt%), w/ S&P2(SC 50 wt%), and w/ S&P2(SC 60 wt%). The curves come from the cycle data in Figure 5.
